# Ashwagandha Root Extract Stabilises Physiological Stress Responses in Male and Female Team Sports Athletes During Pre-Season Training

**DOI:** 10.3390/nu18020230

**Published:** 2026-01-12

**Authors:** Olivia C. Coope, Esteban Otaegui, Manolo Suárez, Alex Levington, Maria Abad-Sangrà, Beth Lloyd, Tilly J. Spurr, Blanca Roman-Viñas

**Affiliations:** 1 Blanquerna School of Health Science, Ramon Llull University, C/Padilla, 326, 08025 Barcelona, Spain; blancarv@blanquerna.url.edu; 2 Club Natació Poble Nou, Carrer de Pallars, 277, Sant Martí, 08005 Barcelona, Spain; 3 Fulham FC Academy, Motspur Park, New Malden, Surrey KT3 6PT, UK; 4 Independent Researcher, 08025 Barcelona, Spain; 5 Faculty of Social and Behavioural Sciences, Leiden University, Wassenaarseweg 52, 2333 AK Leiden, The Netherlands; 6 College Lane, University of Chichester, Chichester PO19 6PE, UK; m.spurr@chi.ac.uk; 7 Faculty of Psychology, Education and Sport Sciences, Ramon Llull University C/Císter, 34, 08022 Barcelona, Spain

**Keywords:** ashwagandha, athletes, pre-season, muscle strength, aerobic capacity, recovery, stress, cortisol, cortisone, randomised controlled trial

## Abstract

**Objectives**: This study investigates the effects of 600 mg/day Ashwagandha root extract on physiological stress biomarkers, perception of recovery, muscle strength and aerobic capacity in team sports athletes during pre-season training, a period associated with elevated cortisol and accumulated training stress. **Methods**: Fifty-six athletes (26.8 ± 4.4 years, 1.74 ± 0.10 m, 79.4 ± 17.3 kg, 11.0 ± 7.1 career years) across rugby, water polo and football were randomly assigned to an Ashwagandha (ASH; *n* = 28, 14 males and 14 females) or placebo (PLA; *n* = 28, 14 males and 14 females) group for 42 days. Salivary biomarkers were assessed after training, muscle strength and aerobic capacity were measured during training, and perception of recovery was evaluated with Hooper Index (HI) the following day. Mixed ANOVA was used to determine group × time interactions and Bonferroni post hoc analyses were conducted for multiple pairwise comparisons. **Results**: In female athletes, salivary cortisol increased significantly in PLA (*p* = 0.001), while recovery parameters such as the overall HI score (*p* = 0.001), Delayed Onset Muscle Soreness (DOMS) (*p* = 0.008) and perception of fatigue (*p* = 0.026) scores improved significantly in ASH. In males, salivary cortisone increased significantly in PLA (*p* = 0.022), while Countermovement Jump (CMJ) improved significantly in ASH (*p* = 0.018). Pull-up performance increased in both PLA (*p* = 0.004) and ASH (*p* < 0.0001) in males. **Conclusions**: Supplementation with 600 mg/day of Ashwagandha root extract for 42 days may stabilise stress biomarkers, improve perception of recovery and enhance muscle strength in team sports athletes during pre-season training. The trial is registered on ClinicalTrials.gov with the ID NCT07041853.

## 1. Introduction

Pre-season training is a pivotal phase in an athlete’s annual cycle, aimed at developing physical capacities, refining technical skills and preparing the body for the competitive in-season [1]. The cumulative load of pre-season training has been associated with changes in cortisol concentrations [2,3], which may impair recovery, disrupt anabolic processes and increase the risk of overtraining or injury [4]. In contact and team sports, these risks are further amplified by the intermittent high-intensity nature of training schedule demands, which often include sprints, collisions and rapid directional changes [5,6,7]. These demands can place strain on both the neuromuscular and endocrine systems, influencing cortisol response [8]. Physiological stress can be evaluated through salivary measurements of cortisol and cortisone: hormones that indicate activity of the hypothalamic–pituitary–adrenal (HPA) axis [9]. Moderate- to high-intensity exercise can provoke increases in circulating cortisol levels [10]. In a study involving female athletes, participation in high-stakes matches was associated with a marked reduction in sleep quality, which coincided with a substantial 354% increase in salivary cortisol concentrations [11]. Elevated post-exercise cortisol levels have been linked to suppressed immune function, increased muscle protein breakdown and disrupted sleep patterns, all of which can impair performance and recovery [12,13].

Consequently, effective recovery strategies during the pre-season are essential to mitigate the impact of elevated physiological stress responses and promote optimal adaptation to training. Typical recovery strategies for athletes involve achieving adequate sleep quality, efficient muscle repair and the restoration of hormonal balance [14,15]. Monitoring perceived recovery can offer valuable insight into an athlete’s readiness to train and compete, encompassing perceptions of stress, fatigue, muscle soreness and sleep quality. Assessing salivary hormones in addition may enable practitioners to better evaluate an athlete’s physiological and psychological adaptation throughout the pre-season phase.

Ashwagandha (*Withania somnifera*) root extract, a traditional adaptogenic herb, is known for its potential to modulate the stress response, enhance physical performance and improve the recovery process in healthy populations [16,17]. Recent systematic reviews and meta-analyses have highlighted the efficacy of Ashwagandha supplementation in clinical trials in reducing systemic stress by having a positive effect on wellbeing, muscle strength and exercise endurance parameters [18,19]. Trials using standardised root extracts have demonstrated significant reductions in cortisol in healthy adults and individuals experiencing mild to moderate stress, with effects observed across a range of doses (125–600 mg/day) and supplementation periods (4–12 weeks). These studies indicate that Ashwagandha may modulate the HPA axis as its mechanism of action by reducing the circulating cortisol and potentially improving stress resilience and sleep quality as a result [20,21].

The bioactive constituents of Ashwagandha include steroidal flavanol glycosides, glycowithanolides, steroidal lactones and phenolics, with more than 12 alkaloids, around 40 withanolides and several sitoindosides reported across its roots, aerial parts and berries [22]. The root extract of the Ashwagandha plant is associated with lower risk of containing, or having negligible levels of, potentially harmful compounds such as withanone and withaferin A that are found in higher content in the leaf of the herb [23,24,25]. These compounds may induce liver toxicity or adverse reactions with overuse [26], despite having been reported to elicit anticarcinogenic effects [27,28,29]. While it is not established, this may explain the adverse results found in research documenting liver injuries following Ashwagandha use [30,31]; the supplements reported are shown to contain the leaf or do not state the amount of withanolides, which are the active compounds that produce the adaptogenic effects. A recent in vitro study using primary human hepatocytes found that ethanolic Ashwagandha leaf extract exhibited dose- and time-dependent hepatocellular toxicity, suggesting possible liver risks at higher concentrations. In contrast, the root extract modulated CYP3A4 activity (an enzyme integral to drug metabolism, metabolising approximately 30–50% of known drugs [32]), indicating potential drug–herb interactions [33]. This suggests individuals taking prescription medications should avoid using it, echoed in a case study that found a patient having involuntary muscle contractions following Ashwagandha supplementation alongside use of 2.5 mg diazepam [34]. However, the safety profile of root-extracted Ashwagandha with a standardised amount of withanolides has been well-documented, with studies supporting its use in healthy participants [35,36,37,38], with one study observing administration over 12 months [39]. Therefore, the chosen investigational product KSM-66 Ashwagandha was obtained from the manufacturer Ixoreal Biomed Inc., Los Angeles, CA, USA. KSM-66 is a commercially available Ashwagandha extract derived exclusively from the root and standardised to the highest concentration. The supplement is produced through a green chemistry method that is devoid of any alcohol or chemical solvents. The product contains a root-only extract from Ashwagandha with >5% of withanolides as estimated by the HPLC method.

There is a lack of research specifically examining the effects of Ashwagandha supplementation in athletes participating in contact and team sports. Team sports athletes experience unique pre-season demands, including repeated high-intensity efforts and rapid changes in direction, which may influence recovery and performance [40]. This population is therefore suitable for evaluating interventions aimed at supporting adaptation and resilience during periods of elevated training stress. The optimal dosage, duration and timing of supplementation remain subjects of ongoing investigation. This study aims to address these gaps by evaluating the effects of 600 mg/day of root-extracted Ashwagandha over a 42-day period on salivary hormone concentrations, as well as perceived recovery and muscle strength in the pre-season period in male and female semi-professional athletes assigned to an academy. Accordingly, the present study examines the effects of Ashwagandha root extract supplementation on physiological stress biomarkers, perceived recovery and muscle strength across the pre-season period in athletes, with the aim of assessing whether an herbal supplement can improve these areas. It is hypothesised that root extract Ashwagandha supplementation, across male and female team sports athletes, will achieve the following: (1) reduce salivary cortisol and cortisone concentrations, (2) improve recovery perception, and (3) enhance muscle strength outcomes, providing a clear, testable framework for assessing its effectiveness in a team sport setting.

## 2. Materials and Methods

### 2.1. Study Design

The study followed a randomised, double-blind, placebo-controlled design over a 6-week period. Participants were recruited through convenience sampling from a sports academy in Barcelona, Spain and included athletes from rugby, water polo and football.

Eligible participants were team sports athletes assigned to a professional sports academy in Barcelona, Spain, aged above 18 years, competing at a sub-elite level and classified as ‘healthy’ or free from disease. Exclusion criteria included active supplementation with other ergogenic aids, pregnancy, allergies to nightshades, use of medications or hormonal contraceptives, to avoid potential interactions of Ashwagandha root extract, which may modulate CYP3A4 enzyme activity involved in drug metabolism [33]. Withdrawal from the study was permitted for participants who requested to exit or failed to complete the required study tests. Participants received a small monetary compensation of EUR 20 per testing session to support compliance and retention throughout the study period. Offering payment to research participants is recognised as an ethically acceptable method of enhancing recruitment and compliance in clinical trials [41]. Participants were randomised using an online (https://www.randomizer.org/ accessed on 13 June 2025) randomisation allocation programme to either the Ashwagandha or placebo group, with equal distribution by sex. The study participants (*n* = 56) were between the ages of 18 and 35 years (26.8 ± 4.4 years, 1.74 ± 0.10 m, 79.4 ± 17.3 kg and 11.0 ± 7.1 years in career) (Table 1).

### 2.2. Procedures

**Figure 1:**
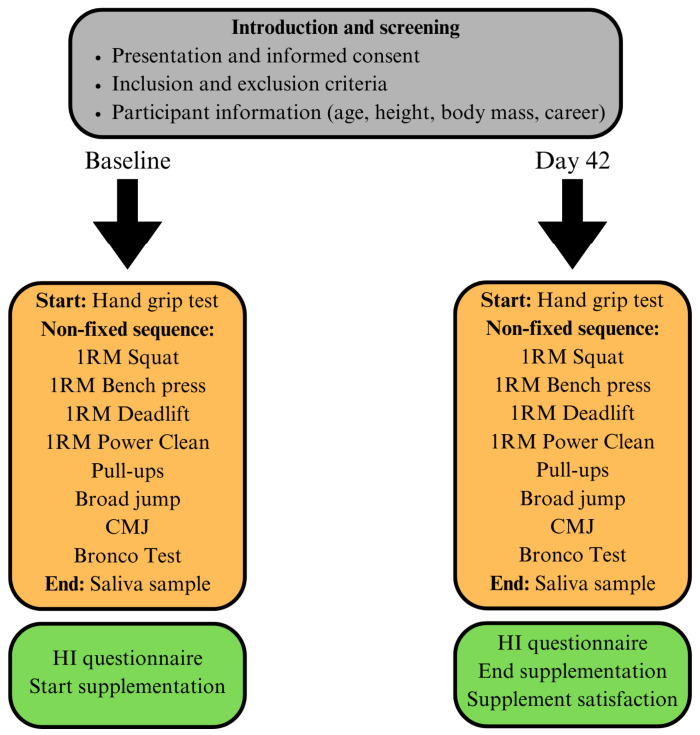
Study timeline. CMJ, countermovement jump; HI, Hooper Index.

At the first visit, all participants received detailed information about the study procedures and were provided with written informed consent. Information about self-reported height and mass and career length of each athlete was collected from the strength and conditioning staff members. The study timeline is presented in Figure 1. For each of the testing sessions, all participants completed assessments evaluating salivary hormone markers, muscle strength, aerobic capacity and perception of recovery using a questionnaire the following day. Data collection for both timepoints took place at the gym of the academy, which is a familiar training ground for all participants.

Training load was recorded by documenting the type, frequency and duration of each session within the academy programme. Sessions combined resistance and conditioning work typical of pre-season preparation with comparable overall demands across sports despite minor differences in exercise content. Strength and conditioning staff supervised all sessions, recording the activities performed, their duration and athlete completion. Each session was designed to elicit a physiological training response consistent with pre-season intensity. Training sessions commenced at 19:30 h and there was a consistent temperature of 28–30 °C with 70–80% humidity.

Saliva samples were collected at the end of every training session using Salivette sample tubes (Sarstedt, Nümbrecht, Germany), documented as a reliable method to evaluate salivary cortisol compared to total and calculated serum cortisol levels [42,43]. Participants were instructed to not eat or drink (except water) at least 30 min prior to sampling. At 21:00 h, each athlete provided a passive saliva sample. Samples were immediately stored on ice before being transferred to a −20 °C freezer for later analysis of hormonal markers. The salivary samples had to be equal to or above 0.5 mL in quantity in order to evaluate cortisol, cortisone, testosterone, DHEA-S and amylase. Stress biomarkers were measured post-training to monitor the acute hormonal response to resistance exercise, as they play a key role in tissue remodelling and adaptation [44]. Assessing cortisol in particular, the primary variable of this study, allows for the quantification of exercise-induced physiological stress and provides insight into the effectiveness and recovery demands of the training protocol.

Recovery and wellbeing were assessed using the Hooper Index (HI), a validated subjective tool designed to capture exercise-induced fatigue in athletes [45]. The HI evaluates four key components: sleep quality, stress, fatigue and muscle soreness, with participants rating each domain on a 1–10 scale, where lower scores indicate better recovery. This method provides a practical and sensitive measure of how athletes perceive their recovery following training. To standardise data collection, the HI was distributed via WhatsApp and completed at 12:00 h on the day following each training session. Menstrual cycle data were self-reported but excluded from analysis. Evidence suggests hormonal fluctuations have minimal effects on strength, performance or recovery, and inconsistent verification methods limit firm conclusions [46]. Although small effects on recovery have been observed in endurance athletes, the menstrual cycle appears to be one of several minor stressors rather than a key factor [47,48].

Maximal strength was determined at the start of the training session with handgrip strength test, measured with the dominant hand using a dynamometer (CAMRY, CA, USA). It is a commonly used tool in sports performance and demonstrates validity and reliability in relation to athlete strength [49]. The validity for the use of a CAMRY dynamometer is documented [50] when comparing against the Jamar dynamometer. Only one maximal attempt was required, as previous work has shown single trials to yield results comparable to the mean of three [51]. Grip span was adjusted individually for comfort and accuracy. Following this measure, participants performed one-repetition maximum (1RM) tests for squat, bench press, deadlift and power clean. Additionally, explosive performance was captured through pull-ups, countermovement jump (CMJ) and standing broad jump These tests are well-established as reliable field indicators of lower-limb power [52]. Aerobic fitness was measured with the Bronco test, which consists of a 1.2 km shuttle run test. The Bronco test shows good validity, with maximum heart rate significantly correlated to match-play heart rate [53]. Testing order was not fixed between participants.

**Figure 2:**
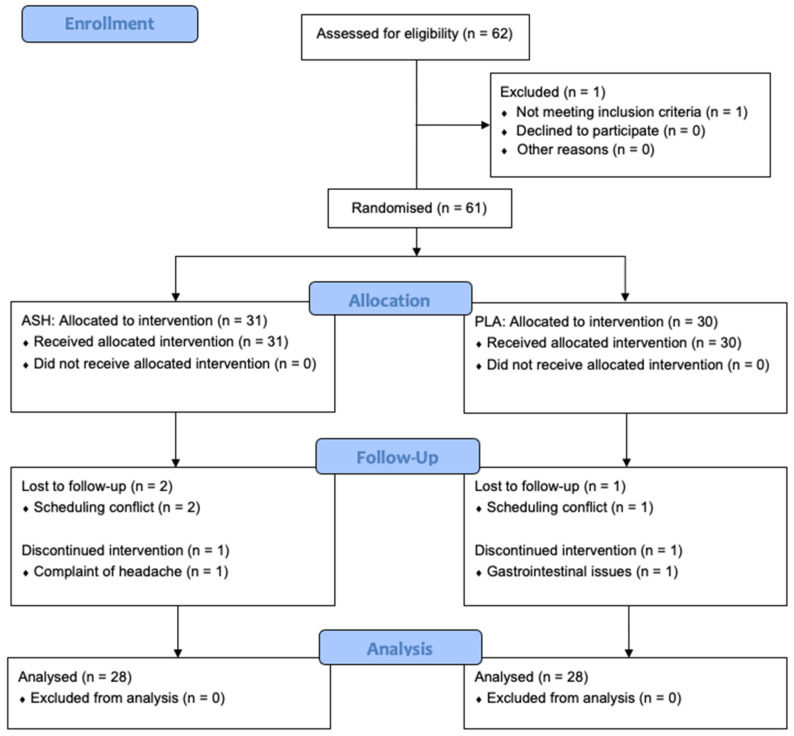
CONSORT diagram. Consolidated Standards of Reporting Trials (CONSORT) diagram. ASH, root extract of Ashwagandha; PLA, placebo.

After the initial baseline measurements, participants commenced a 42-day daily supplementation protocol, receiving either 600 mg/day of the KSM-66 Ashwagandha-root extract (ASH), standardised to >5% withanolides, or 600 mg/day of chickpea flour encapsulated in hydroxypropyl methylcellulose (HPMC) as a placebo (PLA). Researchers were blinded to the allocation. Supplements were taken with dinner and a glass of water, as instructed by the study investigators. Chickpea flour was chosen for its visual similarity to Ashwagandha extract and its gluten-free properties, accommodating participants with potential gluten intolerance. The KSM-66 Ashwagandha extract is certified by Informed Ingredient, a comprehensive anti-doping programme that tests raw materials for banned substances in dietary supplements. Supplement adherence throughout the study was 100% in the remaining participants, monitored through daily check-ins conducted by the research team and academy staff. The monitoring of adverse events was undertaken jointly by the academy staff and research team. In addition, a supplement satisfaction survey was taken at the end of the supplementation period in both the ASH and PLA groups. Scores were taken from a Likert-scale-style survey (1 = very satisfied, 10 = very dissatisfied), inspired by the previous literature that assessed supplement satisfaction of tart cherry after analysing for muscle pain during running [54]. Figure 2 provides a CONSORT (Consolidated Standards of Reporting Trials) flow diagram of the study.

### 2.3. Ethical Committee

The study and the details of the informed consent form were approved by the Research Ethics Committee of the School of Health Sciences of Blanquerna Institute, University Ramon Llull (CER-FCSB) in June 2025 (Approval number: 05-03-2025). The trial was registered prior to data collection on ClinicalTrials.gov with ID NCT07041853.

### 2.4. Statistical Analysis

The required sample size for the study was estimated using G*Power (version 3.1.9.6) and follows the guidelines for sample size estimation in sport and exercise science research by including a formal a priori sample size estimation and rationale [55]. Previous research shows Ashwagandha produces a large effect on symptoms of stress and anxiety in stressed adults, with reported effect sizes of Cohen’s d = 0.8–1.2 [56,57,58]. When converted to ANOVA metrics, this corresponds to an effect size of approximately f = 0.40–0.60. To avoid potentially underpowering the trial, the sample size calculation is conservatively based on a medium effect (f = 0.25). The analysis assumes an effect size of f = 0.25, a significance level of α = 0.05 and power = 0.80, with a correlation of 0.5 among repeated measures. This calculation indicates that 34 participants are needed for statistical validity, with 17 in each of the ASH and PLA groups. The calculation was performed for the overall sample to ensure sufficient power for group-level effects, with results presented separately for men and women.

A 2 × 2 mixed-design ANOVA was employed with Timepoints (Baseline, T1) as the within-subject factor and Condition (PLA, ASH) × Sex (male, female) as between-subjects factors. Variables showing a significant Condition × Time interaction were checked for baseline differences using *t*-tests for normally distributed variables and Wilcoxon tests for non-normal variables. For outcomes with significant baseline differences, ANCOVA was applied using the baseline value as a covariate. Post hoc analyses were performed following the initial analyses and pairwise comparisons were adjusted using the Bonferroni correction to control for the increased risk of false positives, producing the final *p*-value. Supplement satisfaction scores from both the ASH and PLA groups were analysed using Mann–Whitney U tests as scores were on a Likert scale, resulting in ordinal data. For each variable, a single 95% confidence interval (CI) was calculated for the combined sample (ASH and PLA) at each timepoint using the pooled mean and its standard error.

The majority of variables deviated from normality, as indicated by Shapiro–Wilk tests. Data continued to be analysed with ANOVA, a statistical method that is robust to moderate deviations from normality when sample sizes are moderate and equal across groups (*n* = 14 per group/sex) [59].

### 2.5. Processing of Saliva Samples

Saliva samples were stored at −20 °C until analysis. The samples were analysed by Dresden LABservice GmbH (Dresden, Germany). Alpha-amylase was measured using a Genesis RSP8/150 liquid handling system (Tecan, Germany). Samples were diluted 1:625 and 20 µL of diluted saliva or standard were transferred to 96-well microplates. Standards (5.01–326 U/L) were prepared from a Calibrator f.a.s. solution (Roche Diagnostics, Mannheim, Germany). Eighty microliters of substrate reagent were added and plates were incubated at 37 °C. Absorbance at 405 nm was measured before and after a 5 min incubation, and concentrations were calculated using linear regression per plate (GraphPad Prism 4.0c, San Diego, CA, USA). Cortisol was measured after centrifugation of thawed samples at 3000 rpm for 5 min. Concentrations were determined with a high-sensitivity chemiluminescence immunoassay (IBL International, Hamburg, Germany), with intra- and inter-assay coefficients of variation below 8%.

Testosterone, DHEA-S and cortisone were analysed by LC-MS/MS using Shimadzu LC-20AD pumps, an SIL-20AC autosampler, and a CTO-20AC column oven (Shimadzu Corporation, Kyoto, Japan), coupled to an AB Sciex API 5000 triple quadrupole mass spectrometer with an APCI source (AB Sciex LLC, Framingham, MA, USA). Samples underwent on-line solid-phase extraction with a Chromolith SpeedROD RP-18e column (Merck KGaA, Darmstadt, Germany) prior to separation on a Shim-pack XR-ODS analytical column (Shimadzu Corporation, Kyoto, Japan) with a C18 guard cartridge (Phenomenex Inc., Torrance, CA, USA). The system was controlled via Analyst software (v1.5.1), with nitrogen and zero-grade air supplied by a high-purity nitrogen generator. Melatonin was also intended for analysis but degraded during processing and delivery due to light exposure and was therefore not included in the final dataset.

For testosterone, the assay limit of quantification (LOQ) was 1 pg/mL [60]. Three observations in the non-detectable group fell below this threshold. To include these values in the analyses while accounting for the measurement limitation, a common substitution method was applied by replacing each value below the LOQ with half the LOQ (0.5 pg/mL) [61].

A total of 29 values were missing across hormone outcome variables and 32 across the muscle strength variables. Missing data were addressed using multiple imputation by chained equations with predictive mean matching. To preserve the integrity of group-specific distributions, imputations were conducted separately within each treatment–sex subgroup (e.g., placebo-male, placebo-female, supplement-male, supplement-female). Within each subgroup, imputation models included Subject and Timepoints as predictors to account for repeated measurements. For each subgroup, five imputations were generated. Results from these imputed datasets were then pooled and used for subsequent analyses. This was performed to reduce bias and maintain valid inference under the assumption that data were missing at random, consistent with previous applications of multiple imputations in biomarker research [62].

## 3. Results

Significance was set at *p* < 0.05, with data presented as mean ± SD. No significant baseline differences were found between ASH and PLA groups in characteristics (age, height, body mass, career length), as assessed with independent *t*-tests (Table 1). A closely monitored adverse effect was gastrointestinal distress in the case of ingestion, in case there was an undiagnosed allergy of nightshades [63,64]. One participant in the ASH group reported a mild headache. One participant in the PLA group experienced minor gastrointestinal discomfort. In addition, two participants from the ASH group and one from the PLA group discontinued participation due to scheduling conflicts unrelated to the intervention, resulting in 28 participants per group. The *p*-values reported in the table are unadjusted and are presented prior to Bonferroni post hoc correction.

### 3.1. Salivary Hormone Markers

**Figure 3:**
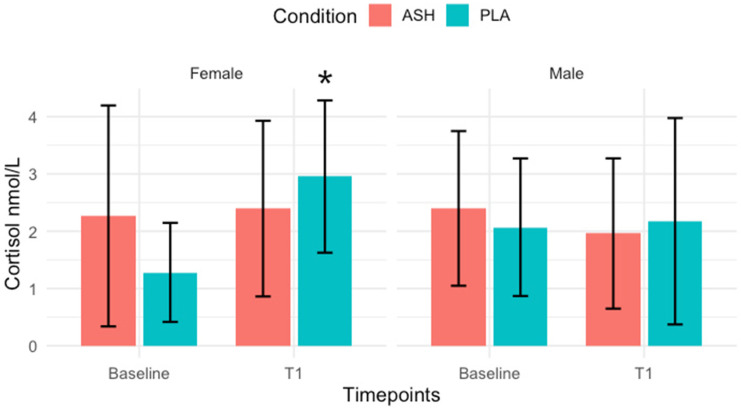
Salivary cortisol. Post-training, night-time salivary cortisol for ASH (red bars) and PLA (green bars) represented as means ± SD. Significant increase (*p* = 0.001) was noted in the PLA group from Baseline to T1 (42 days) following post hoc Bonferroni correction test. Significant change indicated with *.

**Figure 4:**
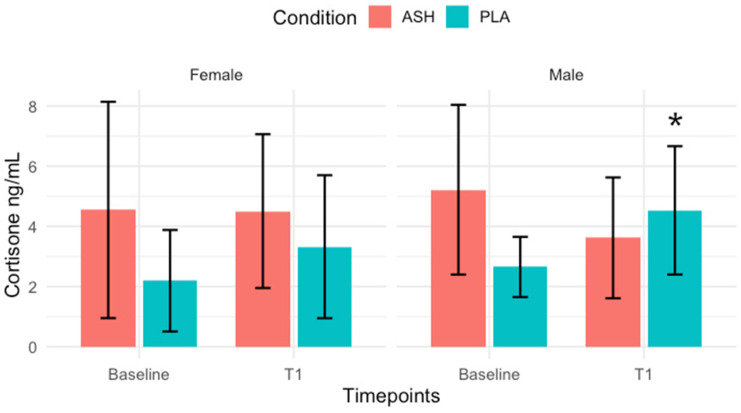
Salivary cortisone. Post-training, night-time salivary cortisone for ASH (red bars) and PLA (green bars) represented as means ± SD. Significant increase (*p* = 0.022) was noted in the PLA group from Baseline to T1 (42 days) following post hoc Bonferroni correction test. Significant change indicated with *.

The full results are shown in Table 2 and Table 3. An analysis of mixed ANOVA revealed a significant group × time interaction for cortisol in females (*p* = 0.010), whereas no significant interaction was observed in males (*p* = 0.393). Post hoc Bonferroni correction test showed a significant increase in cortisol from baseline to 42 days in the PLA female group (*p* = 0.001), shown in Figure 3. Cortisone displayed a significant group × time interaction in males (*p* = 0.0003), with post hoc analysis indicating a significant increase from baseline to 42 days in the PLA male group (*p* = 0.022), shown in Figure 4. Amylase demonstrated a significant interaction in females (*p* = 0.022), although post hoc tests did not reach significance (*p* = 0.267). No significant interactions were observed for testosterone (Males: *p* = 0.915; Females: *p* = 0.615), DHEA-S (Males: *p* = 0.386; Females: *p* = 0.050), or the testosterone-to-cortisol ratio (T/C) (Males: *p* = 0.351; Females: *p* = 0.161).

### 3.2. Perception of Recovery

**Figure 5:**
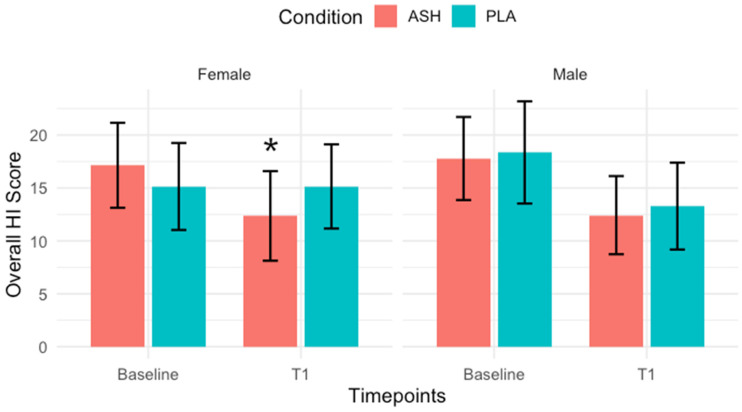
Overall Hooper Index Score. Significant improvement (*p* = 0.001) was noted in the female ASH group from Baseline to T1 (42 days) following post hoc Bonferroni correction test. Significant change indicated with *.

The full results are shown in Table 4 and Table 5 for female and male participants, respectively. No significant changes were observed in the male participants, and significant results regarding recovery were found exclusively in female participants. ANOVA revealed a significant group × time interaction for Overall (accumulated) HI scores in females (*p* = 0.005). Post hoc analysis showed a significant improvement from baseline to T1 in the ASH group (*p* = 0.001), as shown in Figure 5. DOMS also demonstrated a significant interaction (*p* = 0.0002), with post hoc analysis indicating a significant improvement from baseline to T1 in ASH females (*p* = 0.008). Fatigue scores exhibited a significant group × time interaction (*p* = 0.015); post hoc tests revealed a significant improvement from baseline to T1 in the ASH female group (*p* = 0.026).

### 3.3. Muscle Strength and Aerobic Performance

**Figure 6:**
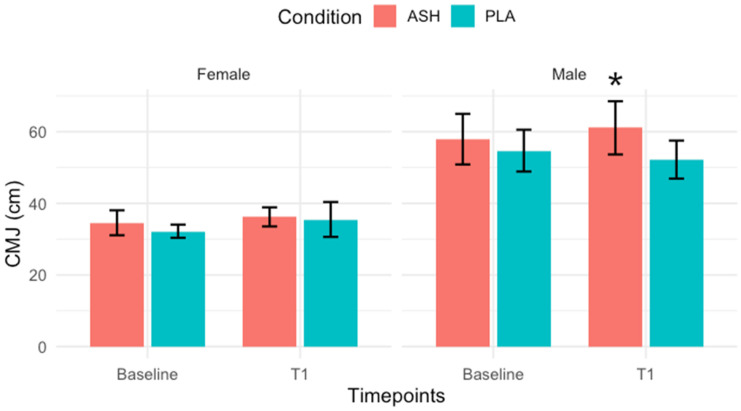
Countermovement jump. Countermovement jump (CMJ) height measured in centimetres (cm) for ASH (red bars) and PLA (green bars) represented as means ± SD. Significant improvement (*p* = 0.018) was noted in the male ASH group from Baseline to T1 (42 days). Significant change indicated with *.

Significant results regarding muscle strength were observed exclusively in male participants. There were no significant changes in the Bronco test for either sex. ANOVA revealed significant group × time interactions for pull-up performance in males; however, a post hoc analysis demonstrated significant increases from baseline in both PLA and ASH male groups (*p* = 0.004 and *p* < 0.0001, respectively). CMJ showed a significant interaction in males (*p* = 0.0003), with post hoc tests indicating a significant increase from baseline to T1 in the ASH male group (*p* = 0.018), as shown in Figure 6. No significant group × time interactions were observed for the other muscle strength variables. The full results are shown in Table 6 and Table 7, for male and female participants, respectively.

### 3.4. Supplement Satisfaction

The ASH group, who supplemented their diet with 600 mg/day of KSM-66 root extract Ashwagandha for 42 days, reported a mean satisfaction score of 3.04 ± 1.1, while the PLA group, who supplemented their diet with a placebo made from chickpea flour, reported a significantly higher score of 4.25 ± 1.2. A Mann–Whitney U test indicated a significant difference between groups (*p* = 0.0004). Lower scores reflect greater satisfaction in the ASH group.

## 4. Discussion

### 4.1. Primary Outcomes

This study is the first to investigate the effects of Ashwagandha root extract supplementation on salivary hormones in semi-professional male and female team sports athletes. Over the 42-day intervention, sex-specific responses were observed, with Ashwagandha appearing to attenuate stress-related hormonal increases seen in the placebo group. Significant group × time interactions were found for cortisol in females and cortisone in males, while amylase showed a trend toward interaction in females. No effects were noted for testosterone, DHEA-S or the testosterone-to-cortisol ratio.

Previous studies have reported a decrease in resting cortisol levels following Ashwagandha [20,21]; however, this present study found that cortisol levels remained stable in the ASH group for females throughout six weeks of pre-season training, a time period where cortisol would typically be elevated without supplementation [65,66]. Only the placebo group exhibited elevated cortisol levels over the same period. This suggests ASH may not blunt the normal physiological stress response but rather help maintain cortisol within an adaptive range, thereby preventing excessive endocrine disruption associated with overtraining [67]. Cortisol plays a dual role during pre-season, supporting energy metabolism and tissue remodelling [68]; however, sustained elevations can impair recovery and performance [69]. Specifically, chronic elevations of cortisol may impair muscle function and sleep quality [70]. Therefore, the stability observed in the ASH group may reflect a more efficient physiological stress regulation, which allows for an effective adaptation to training demands without excessive catabolic strain [71]. A similar pattern was observed for the salivary marker of cortisone in males, with the placebo group showing a significant increase while ASH remained stable. Cortisone represents the inactive metabolite of cortisol [72] and has been shown to increase alongside cortisol during periods of intense training, with changes in the cortisol/cortisone ratio closely reflecting training load, strain, fatigue and alterations in performance, as shown in male rugby players [73]. These findings may indicate that ASH may help to preserve glucocorticoid balance and prevent excessive activation of the HPA axis, leading to better management of the pre-season training burden. This could contribute to more efficient recovery and adaptation by supporting the body’s physiological stress response without inducing prolonged catabolic effects.

The evening, post-exercise (21:00 h) salivary cortisol results observed in this study across both groups fall within, and in some cases below, the expected late-evening physiological range reported in the previous literature. In trained athletes, salivary cortisol concentrations increased from 2.6 nmol/L at the start of pre-season to 5.3 nmol/L after six weeks of resistance training, with samples collected during exercise sessions [74], reflecting the patterns observed in the placebo group of this trial. In comparison, the present study showed similarly modest evening cortisol concentrations, with values remaining stable in the Ashwagandha group compared to increases in the placebo group. This parallel suggests that while Ashwagandha may not directly suppress evening cortisol, it could play a stabilising role under conditions of physical or psychological stress, helping to prevent elevations typically associated with training or competition.

The previous literature supports the use of salivary cortisone as a reliable and stable biomarker of physiological stress [75]. Salivary cortisone displays strong temporal stability and lower day-to-day variability than cortisol, making it suitable for detecting small endocrine changes. It has also been shown that salivary cortisone closely reflects plasma cortisol responses following exercise on a treadmill, suggesting an important role for salivary cortisone as a marker of free cortisol response during exercise [76]. The stability observed in salivary cortisone concentrations in the Ashwagandha group, compared with the significant increase in the placebo group, may indicate that Ashwagandha helped to maintain HPA axis balance under training stress and that the supplement may have attenuated physiological strain across the 42-day period, supporting a consistent endocrine environment. Although attenuating excessive endocrine stress may be beneficial in circumstances, some activation of stress and inflammatory pathways is required to drive muscular and performance adaptations.

An inflammatory response is essential for muscle adaptation and performance gains in sport and has been described as a “mixed blessing” for muscle homeostasis and plasticity [77]. While acute inflammation facilitates tissue repair and strength development, excessive or prolonged inflammation can impede recovery and delay peak performance [78]. Our study observed that 6 weeks of Ashwagandha root extract supplementation did not suppress the typical cortisol response to training, suggesting that it may support the body’s natural inflammatory processes during this critical adaptation phase.

Sex differences in HPA axis regulation may help explain the different responses observed between male and female athletes. Evidence suggests that women may experience greater HPA activation and more persistent physiological changes in response to certain types of stress, whereas men tend to show higher adrenocorticotropic hormone and cortisol responses to performance stressors [79]. Previous research analysing sex-specific difference in the interconversion of cortisol and cortisone in men and women reported that the amount of generated labelled cortisone was significantly higher in men than in women, suggesting greater peripheral conversion of cortisol to cortisone through higher 11β-hydroxysteroid dehydrogenase type 1 oxoreductase activity [80]. This may explain why cortisol increased in females and cortisone increased in males in the placebo group. The stable responses in both Ashwagandha groups suggest that the supplement helped regulate HPA axis activity and minimise stress-related hormonal fluctuations during training.

### 4.2. Secondary Outcomes

Based on the existing evidence regarding ASH supplementation and perception of recovery in athletes [81], it was hypothesised that a 42-day intake of 600 mg/day of Ashwagandha root extract would elicit favourable effects across perceptive recovery markers taken from the HI compared with placebo. There were selectively improved recovery outcomes in female participants. These findings indicate that Ashwagandha may positively influence subjective recovery markers, potentially facilitating adaptation and readiness in female athletes during training periods. The perceptive improvements may be linked to Ashwagandha’s stabilising effect on the HPA axis, as reflected by stable cortisol levels in the ASH group compared with elevated levels in the placebo group. Maintaining cortisol within an adaptive range may support recovery and reduce physiological stress, providing a potential mechanistic explanation for the observed improvements in perceptual markers.

For muscle strength, significant changes were noted in both the male ASH and PLA groups, which would be a typical outcome of the pre-season training period. ANOVA revealed significant group × time interactions for pull-up count; post hoc analyses showed significant increases from baseline in both the PLA and ASH male groups, suggesting a training effect in both conditions. However, there was a significant interaction in only the male ASH group for CMJ, with post hoc tests showing a significant improvement from baseline to T1, indicating a benefit of supplementation on explosive lower-body power. CMJ is widely used as a reliable marker of lower-body power, neuromuscular function and fatigue in athletes [82,83,84]. It is a valid measure for monitoring performance and capabilities in sports such as football, rugby and water polo [85,86,87]. Improvements in CMJ height are moderately to largely correlated with lower-body power in squat exercises, performance in linear sprints and change in direction tasks [88], which may help athletes in pre-season by boosting their ability to perform sport-specific movements. No other physical performance measures displayed significant interactions. The intervention was generally well-tolerated, with one adverse event reported in each group, and participants reported positive responses towards ASH over placebo on the satisfaction questionnaire, indicating good acceptability.

Although this study was not specifically designed to investigate sex differences, it is notable that males and females differed across several outcomes. These differences may reflect physiological factors such as hormonal variations, differences in muscle mass or neuromuscular function and training history. In addition, it is worth noting that, during the trial, participants were exposed to high temperatures (~30 °C) and high humidity (70–80%) during the month of July in a southern European country. Heat stress can increase cortisol and induce a leukocyte response [89]. This may alter the body’s response to training or supplementation. It can also reduce muscle performance, slow down the recovery process, lower exercise capacity and increase feelings of fatigue and muscle soreness [90]. These contextual factors should be considered when interpreting the study’s findings.

Few, but notable, studies in athletes have examined supplements comparable to Ashwagandha for regulating cortisol responses during athletic training. A previous study observed that ingestion of a 6% carbohydrate solution during high-intensity running attenuated post-exercise cortisol elevations compared with placebo in trained marathon runners [91]. Vitamin C supplementation was reported to mitigate the immediate post-race cortisol response following an ultramarathon compared with placebo [92]. Additionally, phosphatidylserine supplementation has shown effects in attenuating exercise-induced cortisol increases, significantly reducing peak and total cortisol concentrations compared with placebo [93]. *Rhodiola rosea*, a recognised adaptogen, has demonstrated potential to enhance endurance performance and reduce fatigue by improving resilience to physiological stress, though its effects on cortisol remain nonsignificant [94]. In a non-athletic participant group, Omega-3 fatty acid supplementation demonstrated lower salivary cortisol during an acute stress challenge compared to placebo [95]. Ashwagandha appears to exert a more consistent effect on maintaining stable cortisol concentrations, supporting its role as an effective modulator of HPA axis activity in the context of exercise.

### 4.3. Limitations and Strengths

There are several limitations to this study. Convenience sampling was used, which helped recruitment but restricts the external validity of the findings beyond the study sample. The lack of a crossover design further limits the ability to control for potential confounders. Dietary intake across participants was not recorded, which is a potential confounding factor as nutritional habits can affect recovery and performance. The observed baseline differences may reflect heterogeneity in the sample, as participants were drawn from three different sport types with varying training backgrounds and performance levels, which may influence the generalisability of the findings. The non-standardised order of assessments across participants is a secondary limitation, although testing conditions were consistent and familiarisation was provided. Salivary hormone samples were collected in the evening to reduce circadian variation; however, individual differences in circadian rhythms remain a potential limitation when interpreting hormonal outcomes. Differences in habitual load, physiology and performance capacities may have contributed to unequal distributions of baseline values despite randomisation. Although several variables deviated from normality, ANOVA was used, which is generally robust in moderating deviations in small, equal-sized groups; however, multiple comparisons may increase the risk of false-positive results. Baseline differences were accounted for by using ANCOVA, including each variable’s baseline value as a covariate. The CONSORT guidelines recommend against formal statistical testing of baseline differences in the domain of sports medicine research [96]. Baseline values in this study are reported, and where baseline differences are present, they are addressed using baseline-adjusted analyses rather than being used to judge group comparability, in line with the updated CONSORT guidance [97].

Additional strengths include the study controls, which involved a randomised, double-blind, placebo-controlled design, recognised as the gold standard for clinical research, which can minimise bias and increase the reliability of findings. Importantly, this is the first study to specifically investigate the effect of Ashwagandha on salivary hormone markers in male and female semi-professional team sports athletes, filling a gap in the literature and offering novel insights into its potential benefits in a competitive sporting context.

The outcomes of this study may provide valuable insights into the practical applications of Ashwagandha supplementation during the pre-season phase for athletes. Highlighting its effects on performance and recovery metrics may inform evidence-based strategies for incorporating Ashwagandha into sports nutrition regimens and, particularly drawing from this present study, in sports characterised by high-intensity intermittent efforts and intense training schedules. While these findings highlight the potential efficacy of Ashwagandha root extract, further research is warranted to refine its role in athletic settings. Future studies may wish to explore optimal dosing strategies, supplementation duration and timing relative to training load. Incorporating crossover designs and longitudinal follow-ups could also enhance the understanding of Ashwagandha’s sustained effects on performance and adaptation throughout the competitive season. It is suggested to incorporate standardised dietary and fluid-intake monitoring in future studies to allow for a clear interpretation of hydration status and its interaction with recovery and performance outcomes. Additionally, future studies could investigate the osmotic properties of Ashwagandha root extract and their potential physiological implications in humans. As this study only compared Ashwagandha with placebo, future research could extend these findings by comparing it with other established non-prohibited ergogenic aids to contextualise its effectiveness.

## 5. Conclusions

In this double-blind, placebo-controlled, randomised trial, 42 days of supplementation with 600 mg/day of Ashwagandha root extract during the pre-season training led to significant improvements compared to the placebo group. Namely, these benefits were sex-specific, with improvements in perception of recovery observed in female athletes and lower-body muscle strength in male athletes. Of particular interest, post-training salivary cortisol in females and salivary cortisone in males remained stable following Ashwagandha supplementation, whereas the placebo group exhibited significant increases. These findings are particularly relevant given that pre-season training has previously been shown to elicit elevations in stress-related hormonal responses.

## Figures and Tables

**Table 1:** Participant characteristics and differences by study group.

	ASH (*n* = 28)	PLA (*n* = 28)	*p*-Value	Female (*n* = 28)	Male (*n* = 28)
Age (years)	26.3 (5.1)	27.3 (3.5)	0.400	27.5 (4.2)	26.1 (4.5)
Height (cm)	175.8 (10.2)	173.4 (9.8)	0.382	166.8 (4.7)	182.3 (7.5)
Body mass (kg)	81.8 (14.5)	76.9 (18.3)	0.280	66.4 (9.6)	92.3 (10.9)
Career (years)	9.6 (6.6)	12.4 (7.4)	0.149	7.3 (6.5)	14.6 (5.8)

ASH = 600 mg daily dose of Ashwagandha root extract; PLA = placebo. Significance values were calculated using independent *t*-tests between groups ASH and PLA and Female and Male.

**Table 2:** Salivary hormone markers in female participants.

Variable	Timepoint	Female ASH (*n* = 14)	Female PLA (*n* = 14)	95% CI	Baseline *p*-Value	Group × Time *p*-Value
Cortisol nmol/L	Baseline Day 42	2.27 (1.93) 2.39 (1.53)	1.28 (0.87) 2.95 (1.33)	(1.82, 2.63)	0.098	0.010 *
Cortisone ng/mL	Baseline Day 42	4.55 (3.59) 4.51 (2.56)	2.19 (1.69) 3.32 (2.38)	(2.92, 4.36)	0.148	0.257
Testosterone pg/mL	Baseline Day 42	10.40 (8.93) 12.72 (8.49)	9.29 (6.85) 9.95 (5.48)	(8.63, 12.55)	0.908	0.615
DHEA-S ng/mL	Baseline Day 42	5.15 (6.89) 3.67 (4.53)	3.31 (3.44) 5.41 (8.24)	(2.82, 5.95)	0.800	0.050
Amylase U/mL	Baseline Day 42	132.84 (84.95) 192.62 (97.23)	199.29 (125.18) 161.10 (134.35)	(142.03, 200.89)	0.114	0.022 *
T/C Ratio	Baseline Day 42	9.17 (10.29) 9.27 (9.79)	12.63 (17.54) 4.18 (3.18)	(5.80, 11.82)	0.629	0.161

Post-training, night-time hormone salivary markers in female participants for 600 mg/day Ashwagandha root extract (ASH) and placebo (PLA) represented as means ± SD. CI = Confidence interval. Baseline *p*-Value was calculated using either independent *t*-tests or Wilcoxon rank sum tests depending on normal distribution. Group × Time *p*-Value was calculated using mixed ANOVA. Significant changes are indicated with *.

**Table 3:** Salivary hormone markers in male participants.

Variable	Timepoint	Male ASH (*n* = 14)	Male PLA (*n* = 14)	95% CI	Baseline *p*-Value	Group × Time *p*-Value
Cortisol nmol/L	Baseline Day 42	2.40 (1.35) 1.96 (1.31)	2.07 (1.20) 2.17 (1.80)	(1.78, 2.52)	0.503	0.393
Cortisone ng/mL	Baseline Day 42	5.22 (2.82) 3.62 (2.01)	2.65 (1.00) 4.53 (2.13)	(3.41, 4.60)	0.005 *¶	0.0003 *
Testosterone pg/mL	Baseline Day 42	70.71 (35.99) 79.45 (20.38)	74.73 (34.05) 81.74 (30.06)	(68.75, 84.56)	0.909	0.915
DHEA-S ng/mL	Baseline Day 42	13.96 (9.98) 12.38 (7.01)	14.88 (13.32) 9.01 (10.03)	(9.86, 5.25)	1.000	0.386
Amylase U/mL	Baseline Day 42	179.57 (134.37) 178.51 (116.41)	145.33 (110.08) 119.21 (110.36)	(124.82, 186.49)	0.468	0.567
T/C Ratio	Baseline Day 42	36.40 (27.88) 58.88 (33.73)	56.21 (62.31) 58.33 (41.04)	(41.17, 63.74)	0.395	0.351

Post-training, night-time hormone salivary markers in male participants for 600 mg/day Ashwagandha root extract (ASH) and placebo (PLA) represented as means ± SD. CI = Confidence interval. Baseline *p*-Value was calculated using either independent *t*-tests or Wilcoxon rank sum tests depending on normal distribution. Group × Time *p*-Value was calculated using mixed ANOVA. Significant changes are indicated with *. ¶ indicates that the significant baseline difference was taken into consideration for the final analysis using ANCOVA.

**Table 4:** Perception of recovery in female participants.

Variable	Timepoint	Female ASH (*n* = 14)	Female PLA (*n* = 14)	95% CI	Baseline *p*-Value	Group × Time *p*-Value
HI Score	Baseline Day 42	17.14 (4.02) 12.36 (4.24)	15.14 (4.11) 15.14 (3.98)	(13.81, 16.08)	0.204	0.005 *
DOMS	Baseline Day 42	4.07 (1.33) 2.57 (1.34)	2.43 (1.16) 3.50 (1.95)	(2.73, 3.56)	0.004 *¶	0.0002 *
Sleep	Baseline Day 42	3.86 (2.11) 3.21 (1.89)	3.71 (1.98) 3.50 (2.31)	(3.04, 4.10)	0.962	0.437
Stress	Baseline Day 42	5.00 (2.39) 3.57 (1.95)	5.29 (2.55) 4.21 (2.46)	(3.89, 5.14)	0.762	0.681
Fatigue	Baseline Day 42	4.21 (1.72) 3.00 (1.30)	3.71 (1.54) 3.93 (1.33)	(3.32, 4.11)	0.425	0.015 *

The perception of recovery scores the day after a training session, taken from the Hooper Index (HI) in female participants for 600 mg/day Ashwagandha root extract (ASH) and placebo (PLA) represented as means ± SD. CI = Confidence interval. Baseline *p*-Value was calculated using either independent *t*-tests or Wilcoxon rank sum tests depending on normal distribution. Group × Time *p*-Value was calculated using mixed ANOVA. Significant changes are indicated with *. ¶ indicates that the significant baseline difference was taken into consideration for the final analysis using ANCOVA.

**Table 5:** Perception of recovery in male participants.

Variable	Timepoint	Male ASH (*n* = 14)	Male PLA (*n* = 14)	95% CI	Baseline *p*-Value	Group × Time *p*-Value
HI Score	Baseline Day 42	17.79 (3.93) 12.43 (3.69)	18.36 (4.83) 13.29 (4.10)	(14.20, 16.73)	0.734	0.863
DOMS	Baseline Day 42	4.00 (1.57) 2.50 (1.22)	4.36 (1.55) 3.14 (1.41)	(3.09, 3.91)	0.550	0.617
Sleep	Baseline Day 42	3.21 (1.89) 3.57 (1.65)	4.21 (2.12) 3.36 (1.95)	(3.54, 4.64)	0.242	0.219
Stress	Baseline Day 42	4.21 (0.97) 3.14 (0.77)	4.57 (1.83) 3.00 (1.36)	(3.36, 4.11)	0.723	0.377
Fatigue	Baseline Day 42	4.36 (1.50) 3.21 (1.42)	5.21 (1.76) 3.79 (1.31)	(3.71, 4.57)	0.178	0.690

The perception of recovery scores the day after a training session, taken from the Hooper Index (HI) in male participants for 600 mg/day Ashwagandha root extract (ASH) and placebo (PLA) represented as means ± SD. CI = Confidence interval. Baseline *p*-Value was calculated using either independent *t*-tests or Wilcoxon rank sum tests depending on normal distribution. Group × Time *p*-Value was calculated using mixed ANOVA.

**Table 6:** Muscle strength and aerobic performance in male participants.

Variable	Timepoint	Male ASH (*n* = 14)	Male PLA (*n* = 14)	95% CI	Baseline *p*-Value	Group × Time *p*-Value
1RM Squat (kg)	Baseline Day 42	192.14 (24.24) 221.07 (18.31)	159.64 (29.64) 176.79 (30.10)	(178.49, 196.33)	0.004 *¶	0.111
1RM Bench Press (kg)	Baseline Day 42	116.07 (11.12) 123.21 (7.50)	101.96 (19.76) 105.71 (13.28)	(107.61, 115.87)	0.014 *¶	0.413
1RM Deadlift (kg)	Baseline Day 42	179.29 (21.74) 195.36 (13.93)	176.43 (24.05) 188.57 (23.49)	(179.16, 190.66)	0.208	0.477
Bronco Test (s)	Baseline Day 42	348.64 (16.05) 340.14 (18.00)	347.14 (25.99) 335.57 (16.68)	(337.68, 348.06)	0.856	0.584
1RM Clean Lift (kg)	Baseline Day 42	95.00 (11.44) 97.86 (9.14)	83.21 (17.28) 83.57 (20.42)	(85.65, 94.17)	0.050	0.441
Broad jump (cm)	Baseline Day 42	245.43 (14.67) 248.64 (14.63)	244.79 (13.80) 242.71 (15.00)	(241.65, 249.14)	0.117	0.207
CMJ (cm)	Baseline Day 42	57.93 (7.07) 61.07 (7.43)	54.71 (5.84) 52.21 (5.31)	(54.61, 58.35)	0.201	0.0003 *
Hand grip (kg)	Baseline Day 42	56.21 (7.70) 57.76 (6.53)	53.64 (9.58) 53.31 (9.71)	(53.02, 57.45)	0.728	0.297
Pull-ups	Baseline Day 42	14.07 (3.93) 18.50 (2.88)	12.43 (3.55) 14.57 (3.20)	(13.84, 15.94)	0.604	0.007 *

Muscle strength in male participants for 600 mg/day Ashwagandha root extract (ASH) and placebo (PLA) represented as means ± SD. CI = Confidence interval; 1RM = One-repetition maximum; kg = kilogrammes; cm = centimetres; CMJ = Countermovement jump. Baseline *p*-Value was calculated using either independent *t*-tests or Wilcoxon rank sum tests depending on normal distribution. Group × Time *p*-Value was calculated using mixed ANOVA. Significant changes are indicated with *. ¶ indicates that the significant baseline difference was taken into consideration for the final analysis using ANCOVA.

**Table 7:** Muscle strength and aerobic performance in female participants.

Variable	Timepoint	Female ASH (*n* = 14)	Female PLA (*n* = 14)	95% CI	Baseline *p*-Value	Group × Time *p*-Value
1RM Squat (kg)	Baseline Day 42	144.29 (15.05) 148.57 (14.60)	114.29 (14.53) 123.57 (14.47)	(127.38, 137.98)	0.0002 *¶	0.519
1RM Bench Press (kg)	Baseline Day 42	48.57 (5.86) 50.54 (6.29)	43.75 (3.50) 46.25 (3.89)	(45.83, 48.72)	0.014 *	0.821
1RM Deadlift (kg)	Baseline Day 42	94.64 (4.14) 93.57 (4.13)	87.86 (9.55) 91.43 (9.08)	(89.92, 93.83)	0.080	0.229
Bronco Test (s)	Baseline Day 42	324.86 (28.41) 326.43 (25.95)	403.29 (53.24) 410.14 (47.71)	(351.28, 381.08)	0.0007 *¶	0.811
1RM Clean Lift (kg)	Baseline Day 42	37.50 (2.59) 37.14 (2.57)	24.64 (1.34) 23.57 (2.34)	(28.87, 32.56)	<0.0001 *¶	0.533
Broad jump (cm)	Baseline Day 42	195.14 (17.97) 156.14 (1.70)	198.14 (15.37) 157.14 (3.21)	(170.54, 182.75)	<0.0001 *¶	0.732
CMJ (cm)	Baseline Day 42	34.57 (3.48) 36.21 (2.67)	32.21 (1.85) 35.50 (4.88)	(33.67, 35.58)	0.098	0.336
Hand grip (kg)	Baseline Day 42	32.94 (4.23) 33.48 (5.41)	33.54 (4.67) 33.95 (4.52)	(32.27, 34.68)	0.728	0.899
Pull-ups	Baseline Day 42	2.00 (1.24) 3.29 (0.83)	0.64 (1.08) 1.14 (1.23)	(1.38, 2.15)	0.002 *¶	0.191

Muscle strength in female participants for 600 mg/day Ashwagandha root extract (ASH) and placebo (PLA) represented as means ± SD. CI = Confidence interval; 1RM = One-repetition maximum; kg = kilogrammes; cm = centimetres; CMJ = Countermovement jump. Baseline *p*-Value was calculated using either independent *t*-tests or Wilcoxon rank sum tests depending on normal distribution. Group × Time *p*-Value was calculated using mixed ANOVA. Significant changes are indicated with *. ¶ indicates that the significant baseline difference was taken into consideration for the final analysis using ANCOVA.

## Data Availability

The datasets generated and analysed during the current study are available from the corresponding author upon reasonable request. Access may be granted for the purposes of academic research, subject to any ethical or confidentiality considerations.
